# Digital technology dilemma: on unlocking the soil quality index conundrum

**DOI:** 10.1186/s40643-020-00359-x

**Published:** 2021-01-10

**Authors:** Vincent de Paul Obade, Charles Gaya

**Affiliations:** 1 BioResource and Agricultural Engineering Department, Cal Poly San Luis Obispo, 1 Grand Ave, San Luis Obispo, CA USA; 2 Department of Geomatic Engineering and Geospatial Information Systems, Jomo Kenyatta University of Agriculture and Technology, Juja, Kenya

**Keywords:** Accuracy, Digital mapping, Soil quality, Spatial interpolation

## Abstract

Knowledge of the interactions between soil systems, management practices, and climatic extremes are critical for prescription-based sustainable practices that reduce environmental pollution/footprints, disruption of food supply chains, food contamination, and thus improve socio-economic wellbeing. Soil quality status and dynamics under climate change present both a hazard which may not be remedied by simply adding chemicals or improved by crop varieties, and an opportunity (e.g., by indicating impact of a shift in land use) although the specifics remain debatable. This entry not only revisits the science of soil quality determination but also explicates on intricacies of monitoring using big data generated continuously and integrated using the “internet of things.” Indeed, relaying credible soil quality information especially for heterogeneous soils at field scale is constrained by challenges ranging from data artifacts and acquisition timing differences, vague baselines, validation challenges, scarcity of robust standard algorithms, and decision support tools. With the advent of digital technology, modern communication networks, and advancement in variable rate technologies (VRT), a new era has dawned for developing automated scalable and synthesized soil quality metrics. However, before digital technology becomes the routine tool for soil quality sensing and monitoring, there is need to understand the issues and concerns. This contribution not only exemplifies a unique application of digital technology to detect residue cover but also deliberates on the following questions: (1) is digital agriculture the missing link for integrating, understanding the interconnectivity, and ascertaining the provenance between soil quality, agronomic production, environmental health, and climate dynamics? and (2) what are the technological gaps?
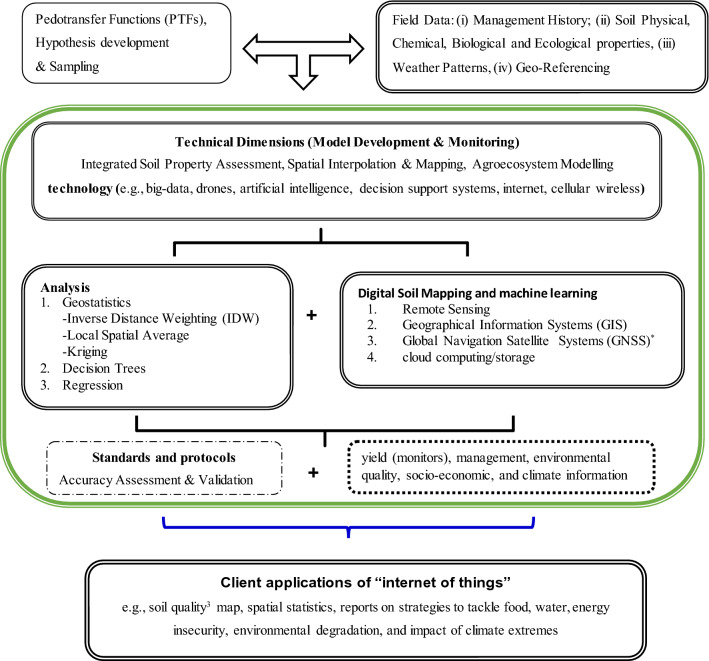

## Introduction

**Fig. 1:**
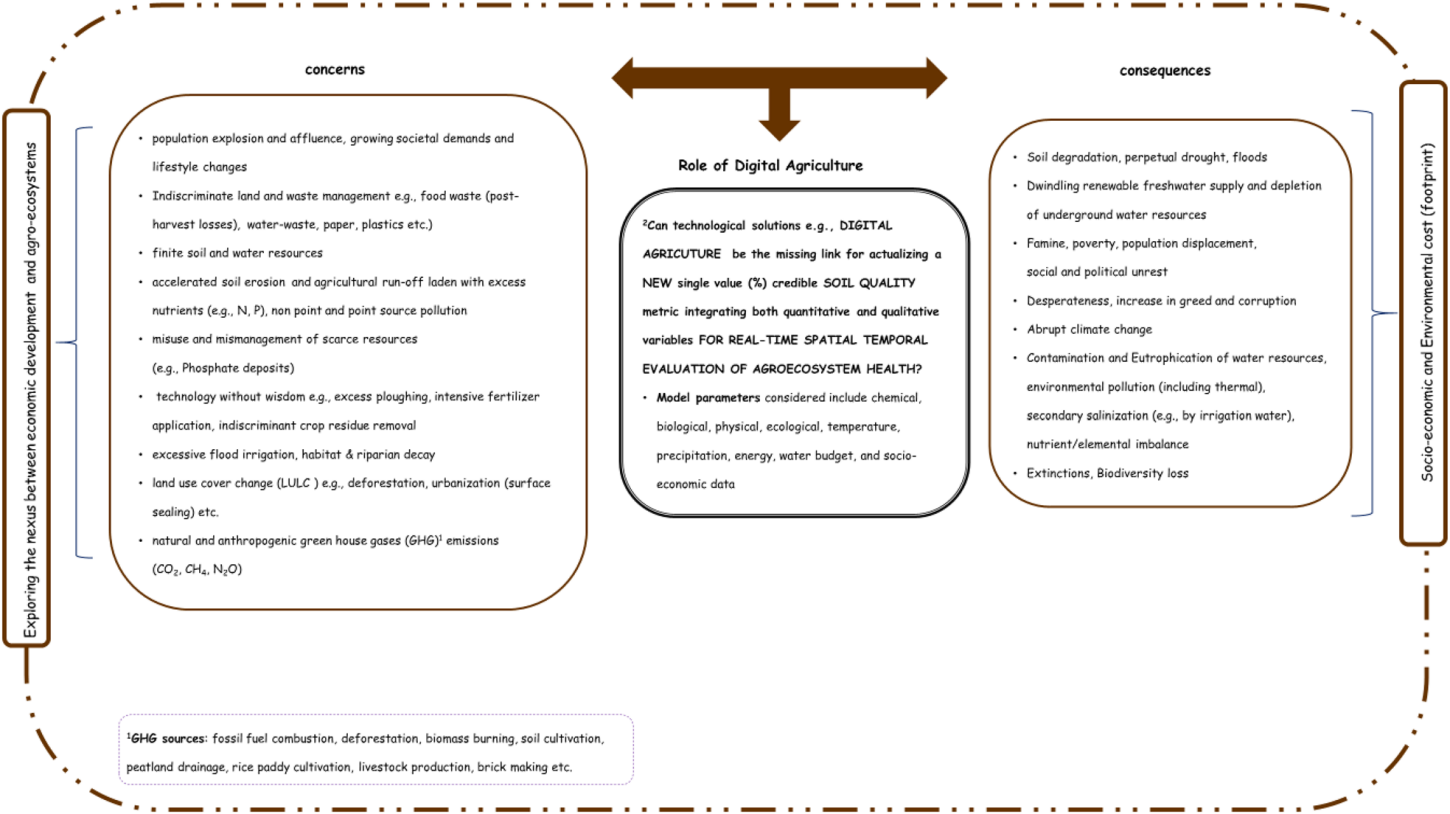
The synergism between socio economic development, agroecosystems and environmental footprint. ^2^The more complicated the problem e.g., soil quality determination, the more data (big-data) required (Modified from Lal (2013), Wyckhuys et al. (2018))

The manifold risks created by pollution, landslides, drought, and pandemics (e.g., COVID-19 in which recovery rates hypothetically correlate with healthy diet and thus to soil quality, because soils with optimal nutrients, water and air produce healthy crops) are aggravated by the skyrocketing human population, lifestyle changes, and inapt technology use (Gleick and Palaniappan 2010; Landrigan et al. 2018; Schiefer et al. 2016; Venegas-Li et al. 2019). This illustrates the pressing need for proactive and strategically targeted land management, for instance, to alleviate the undernourishment of over 810 million people globally (Abbas et al. 2013; de Paul Obade et al. 2014; Lal 2018, 2020; Lal et al. 2020; Landrigan et al. 2018; Paz-Ferreiro and Fu 2016). Despite the much-heralded technological revolution, framing scientific knowledge for sustainable intensification defined as optimizing productivity per unit input of land, with less water, fertilizer, energy, labor, time, and smaller environmental footprint, attainable through minimizing losses and increasing soil, water, and nutrient use efficiency, remains challenging (Arshad and Martin 2002; Bouma and McBratney 2013; de Paul Obade and Moore 2018; Lal 2009a, b; Power 2010; Stockmann et al. 2013). This is attributable to (i) the absence of a standard soil quality baseline because the soil is a multifunctional medium that is spatially heterogeneous and varies temporally, and (ii) the absence of a universal soil quality metric, making soil quality monitoring challenging (de Paul Obade and Lal 2016b; Ohlson 2014). Besides, the impact of exogenous factors, such as climate extremes on soil systems, remain vague (McBratney et al. 2014; Stockmann et al. 2014). For brevity, climate entails averaging temperature, precipitation, humidity, wind velocity, radiation, and cloud cover over approximately 30 years to predict future patterns, yet weather represents these factors on a daily basis (Lal 2013). Climatic extremes impact societies negatively and positively, though the negatives are of most concern. For instance, the climatic disasters in the United States of America (U.S.A) since 1980 have resulted in damages exceeding $ 1.8 trillion, with the 2012 drought alone accounting for agricultural losses of over $ 30 billion (Ndehedehe et al. 2019; NOAA 2020). Notwithstanding, 20% (i.e., ≥ 10 million people) of global fatalities are attributed to consequences of adverse climatic effects, such as flooding and related soil- and water-borne diseases (Landrigan et al. 2018). Figure 1 epitomizes the nexus between soil quality, socio-economics, environmental costs, and digital technologies pertinent for (a) assessing regulatory compliance and restoration plans for destroyed properties and (b) formulating scientific knowledge to gauge socio-economic safety nets.

The technological implications of developments in the agricultural industry and related soil quality impacts need to be understood. Other than efficient engines and rural electrification, the tsunami of continuous integrated data and information sharing initiatives offered by digital technology, here-in referred to as the “internet of things,” collectively support zoning and monitoring of agricultural fields to inform policy (Bentley et al. 2019; Dumont et al. 2018; Fleming et al. 2018; Schiefer et al. 2016; Weersink et al. 2018; Zeraatpisheh et al. 2020). The “internet of things” involves data agglomeration captured using sensors, scaled and synthesized into information using machine learning software, and disseminated through the internet. Because soil quality spatially varies with depth, nutrient cycling dynamics, and leaching, yet impacts soil functions and ecosystem services (i.e., habitat provision, biological regulation, water quality, pest and disease control, pollution control, biomass production, etc.), a wholistic understanding of the soil system vis-à-vis environmental health is critical to guide targeted scientific-based policy. Whereas a *top*-*down* approach used by governmental agencies suffices for monitoring large spatial extents (e.g., non-point pollution sources), the *bottom*-*up* approach applied generally in local or small areal extents relies heavily on input from local stakeholders.

An overview of recent agricultural technology marvels include (i) autonomous robots which can be deployed to optimize output through precision agriculture rather than traditional uniform soil management, and for weed removal; (ii) multispectral cameras to gather information on soil and crop health which can be relayed instantaneously through cellular devices; and (iii) microsensors operating from unmanned aerial vehicles (UAVs) or drones to provide infra-red (IR) imagery useful for pin-pointing unhealthy vegetation (Dash et al. 2017; Kyratzis et al. 2015; Rodriguez-Moreno et al. 2017). Although vertical farms occupy less space, sometimes practical without soil, their impediment is the exorbitant energy costs, because artificial light, specifically blue and red light to optimize photosynthesis is constantly required (Pigford et al. 2018). In vertical farms, sensors can be used to assay and replicate in-house climate, a technological development applicable in reducing CO_2_ emissions in the agricultural industry. Researchers have monitored the animal health dynamics vis-à-vis soil quality and by proxy vegetation health, through fitting smart collars on animals to assess weight and muscle developments (Li et al. 2018; Saravanan and Saraniya 2018). Similarly, poultry movements have been tracked using 3-dimensional (3D) cameras to analyze behavior and diagnose problems (Colles et al. 2016; Mc Inerney et al. 2011; Nakarmi et al. 2014). In aquaculture, artificial pond ecosystems have been developed to reduce soil and water pollution (Toni et al. 2019; Watanabe et al. 2002). These ponds generate no waste because bacteria recycle nutrients and even produce electric power. Other innovative yet prudent measures supporting healthy diets while maintaining environmental quality include reduced consumption of heavily processed foods that generate wastes which become pollutants upon indiscriminate disposal (Dumont et al. 2018; Lal et al. 2020).

As pointed out in Fig. 1, mismanagement, for instance, broadcasting excessive fertilizer on soil surfaces having high erosive or leaching potential, pollutes surface and groundwater (Andrews and Carroll 2001). Although sewage sludge increases soil organic matter (SOM), the heavy metals contained therein are toxic (Nortcliff 2002). Excessively tilled soils left bare are prone to erosion, acidity, and degradation, yet mineral weathering and leaching enhance soil acidity, thereby adversely impacting soil quality through (i) increased concentration of toxic elements (e.g., aluminium and manganese) and (ii) reduced availability in the root zone of buffering plant nutrients (e.g., Ca) (Arnold et al. 2012; Lal 2013; Mattikalli and Richards 1996). Alternately, soil salinity lowers productivity and even damages infrastructure, because of accretion of Na^+^, Cl^−^, Mg^2+^, and SO_4_^2−^ ions, an occurrence exacerbated in soils with poor drainage, or rising groundwater table (Andrews et al. 2003; Broders et al. 2009; He et al. 1993; Laurent and Ruelland 2011; Manandhar and Odeh 2014; Ngo-Mbogba et al. 2015; Yemefack et al. 2006). Fertile soils play a critical role in supporting ecosystem services, such as nutrient cycling, water purification, habitat/biodiversity conservation, biomass production, and climate regulation (Bünemann et al. 2018; de Paul Obade and Lal 2016b; Doran and Parkin 1994; Lal 2018; Taylor et al. 2010); thus, ecosystem services may serve as proxy indicators of soil quality. In the same vein, the soil organic Carbon (SOC) is inextricably linked to soil quality because it supports ecosystem services (Batjes 2011; Ketterings and Bigham 2000; McBratney et al. 2014; Stockmann et al. 2013).

Soil quality is assayed (i) qualitatively, for instance, visually by using the Munsell color chart where darker soils with high organic matter are considered of superior quality or (ii) quantitatively by measuring the soil physical, chemical, and biological attributes (de Paul Obade and Lal 2013, 2014a, b; Staff 1951). For downstream scientific applications, soil attributes can be synthesized into a Soil Quality Index (SQI) (Bünemann et al. 2018; de Paul Obade and Lal 2016a, b; Wienhold et al. 2004). Although site specific soil quality information is critical for understanding soil systems, or identifying key sustainable practices, a universal SQI model fitting all ecoregions remains elusive (de Paul Obade and Lal 2016a), partly because of assumptions (e.g., forest soils are hypothetically considered to be of high quality compared with cultivated soils), introducing uncertainty and inconsistency. Further, a common problem in strategically managing soil quality issues is scarcity of up-to-date accurate soil quality information relayed in real time. The novelty of this contribution, therefore, is that it exposits the potential of digital technology in assaying and rapidly disseminating information on soil quality dynamics.

Analyzing soil quality using traditional “walk in the field” survey and laboratory methods can be a daunting task, that is, labor, time, and cost intensive especially for data collected over a large areal extent (Guo and Gifford 2002; Venegas-Li et al. 2019; West and Post 2002). Besides, the laboratory determination of SOC by chromate oxidation or “wet combustion” method not only releases toxic wastes but can generate inaccurate data because of the incomplete oxidation of SOM, whereas the dry combustion method is expensive and slow. Alternately, loss-on-ignition method, though affordable, is unreliable because some unaccounted mineral fractions are also decomposed at high temperatures (Bai et al. 2018; Batjes 2011; Nelson and Sommers 1996).

**Fig. 2:**
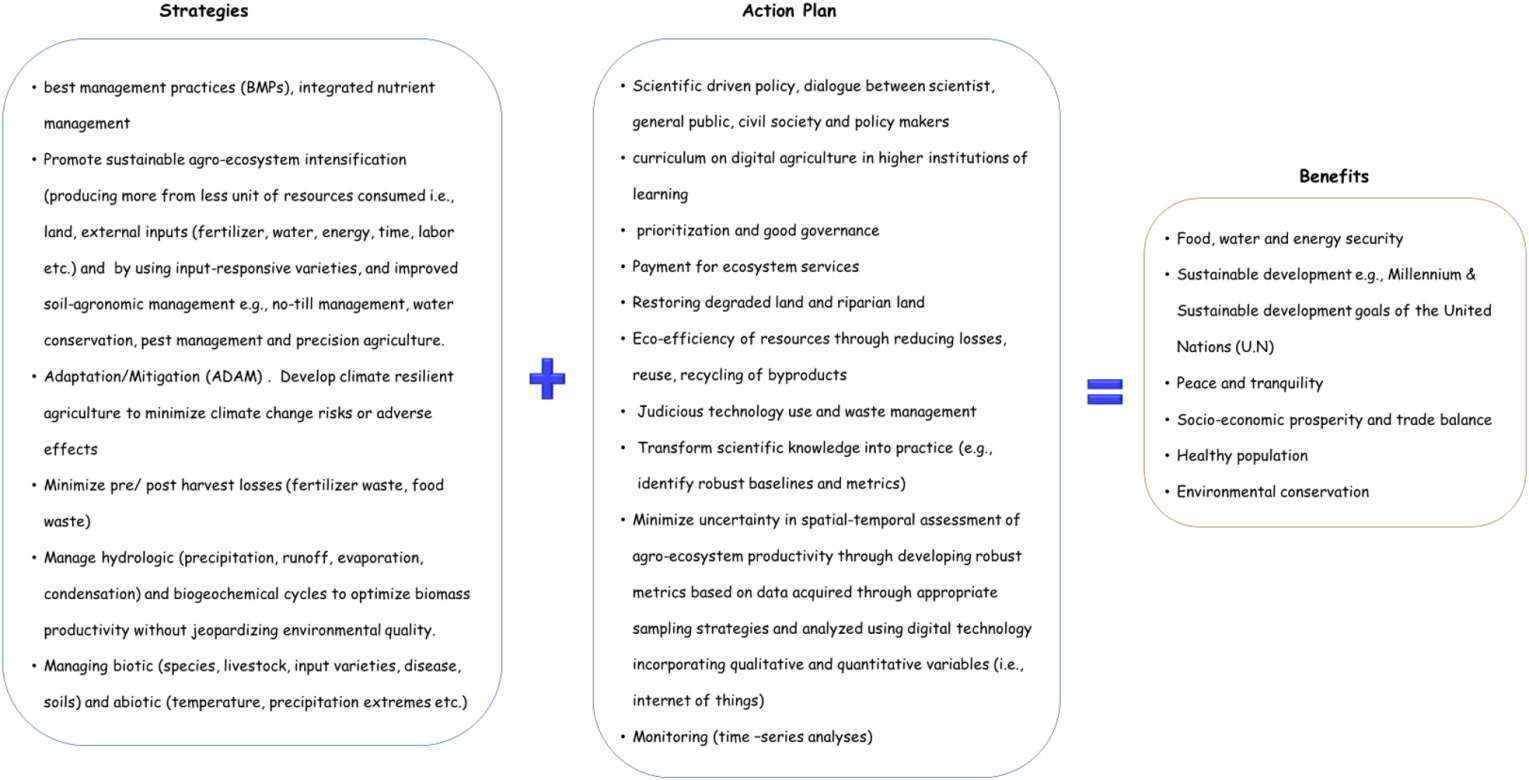
A schematic illustration of the opportunities implementable by digital agriculture technologies (Modified from Lal (2013), de Paul Obade and Lal (2013))

Knowledge on agroecosystem productivity vis-à-vis soil quality dynamics are currently scattered, patchy, and largely inconsistent, making it challenging for end users to understand, prioritize strategies underpinning development, or even apply in policy formulation. Agroecosystem monitoring requires accurate, verifiable baseline information which dictate the methodology and technical expertise equal to this task (Fig. 2). Thus, digital technologies provide the best practical option; however, for these technologies to take root, scientific breakthroughs supported by transformation in educational curricula are required (Schiefer et al. 2016). In this era of online learning, innovative laboratory and field practicals should be incorporated in scientific and technical training programs to produce graduates with hands-on experience. That said, it is reasonable to suggest “wise technology use,” the creation and financing of innovation niches focusing on digital agriculture to generate high-quality scientific research and produce “organic” rather than “academic” intellectuals. Organic intellectuals are critical thinkers who create new ideas, actualize inventions that improve societal wellbeing, whereas academic intellectuals follow the status quo. Sustainable solutions should be all inclusive involving all stakeholders, that is, policymakers, scientist, and general public. Under the hypothesis that digital technology distinguishes managed from unmanaged agroecosystems, this paper exposits on digital technology tenets, opportunities, and limitations for relaying synthesized soil quality information to enhance extension delivery and inform policy.

## Opportunities for digital mapping technology

The increased accessibility to variable rate technologies (VRTs), geospatial data, and communication tools offers new opportunities to ask and answer new questions that were impossible to fathom in the past due to resource limitations and scattered initiatives (Grunwald 2009; Herrick et al. 2017; Keskin and Grunwald 2018; Keskin et al. 2019; Khanal et al. 2018). Of relevance here is the innovation opportunities in digital technology for creating a credible universal digital SQI applicable in all ecoregions. Although digital technology is revolutionizing the agricultural sector by, for instance, generating yield maps in real time, the implications in soil quality determination, human health and disease monitoring (e.g., COVID-19 which by proxy is correlated with soil quality which determines nutrient intake in the food chain and thus human health and antibodies), environmental conservation, harvest planning, cash-flow-budgeting or insurance benefits, and overall costs of this transformation remain fuzzy (Weersink et al. 2018).

The cornerstone of the digital technology concept is the integration of tools and information systems. For instance, the Geographical Information Systems (GIS) integrates and overlays datasets from diverse sources that are statistically analyzed to generate information on in-field soil and crop-yield variability. Examples of databases with georeferenced soil information include SOTER and WISE (Batjes 2011; Minasny and Hartemink 2011). GIS applications can screen out, prioritize, and rank significant model attributes or driving forces influencing soil quality dynamics (Grunwald 2009). An overview of these soil quality attributes, abbreviated as *scorpan* include (1) “*s” representing* soil attributes at a point; (2) “*c”* for climatic properties at a point; (3) “*o” for o*rganisms; (4) “*r” acronym for* topography including terrain attributes and classes, such as slope, aspect, area, and direction; (5) *p is the* parent material, including lithology; (6) *a is the a*ge or time factor; and (7) *n represents* spatial or geographic position (McBratney et al. 2002). Apart from the requirement of skilled analysts, GIS is no panacea and produces unreliable results when data formats are inconsistent (Cohen et al. 2007; Diek et al. 2019; Nocita et al. 2013). Further, GIS maps are usually generalized for clarity; thus, some measurements may not reflect accurate ground position, for instance, spot heights (point features) are magnified for display purposes.

Because detailed information can instantaneously be relayed digitally, the United Nations considers digital technologies to be viable in actualizing Agenda 2 of the sustainable development goal, which focusses on hunger elimination (U.N. 2019). Besides, governance may be improved through rapid information dissemination and decisions on volatile issues, such as judicious water and fertilizer management (U.N. 2019; Weersink et al. 2018). Notwithstanding, management efficiency is boosted by the integrated systems (Herrick et al. 2017; Wyckhuys et al. 2018).

As digital technology continues to take root, strategies are required not only to tackle emerging challenges but also to minimize negative feedbacks and risks especially with regard to improved technological efficiency which may drive unemployment (U.N 2019; Weersink et al. 2018). Concerns associated with the paradigm shift to digital technology include (i) controversies and fanaticism regarding data manipulation and security; (ii) exorbitant development and operational costs that can damage equipment when connecting different technological systems all of which are rapidly evolving; (iii) intentional or nonintentional accidents from spyware or malwares; (iv) transforming beliefs, attitudes, and training users; and (v) data ownership, privacy issues, and potential criminal data misuse (Bentley et al. 2019; Wyckhuys et al. 2018).

## Digital systems and machine learning

**Fig. 3:**
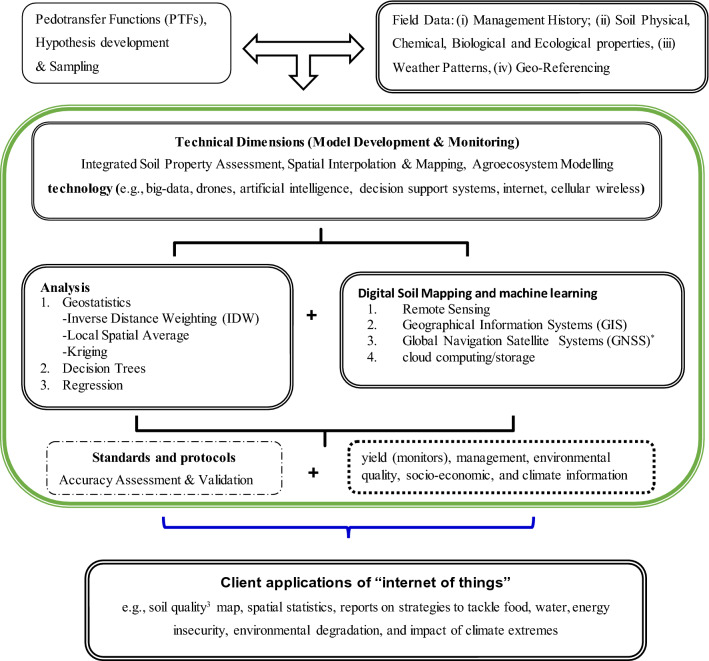
Schematic of integrated digital technology system synonymous with “internet of things” for design of metric to estimate and predict soil quality to guide agroecosystem management. ^*^Global Navigation Satellite Systems include the Global Positioning System (GPS), GLONASS, Galileo, Beidou and other systems. ^3^Soil quality determined by integrating physical, chemical, biological and ecological soil attributes

Dealing with the potential disconnect between policy and science to tackle agroecosystems challenges outlined in Fig. 1 calls for integration of multidisciplinary technologies. Traditionally, conventional “walk in the field” surveys and photogrammetry were utilized in mapping which generated dated information. The advent of “internet of things” that integrates field, global navigational satellite system (GNSS) position data, remotely sensed data, and real-time information gleaned from the internet have generated renewed interest in real-time revision and dissemination of comprehensive, otherwise referred to as “wall to wall” georeferenced information. This section articulates overlapping scientific digital systems, data, and critical analysis for monitoring agroecosystems (Fig. 3).

### Sampling and synthesis

In scenarios where a new model or technique is statistically proven to synthesize and precisely epitomize realistic scenarios, the conventional methods are replaced. Significant environmental attributes can be screened and ranked hinged on robust repeatable experimental designs. Theoretically, sampling predicts values of unsampled location based on a data subset, or observation that statistically estimates characteristics of the whole dataset (Goovaerts 1999). From a practical standpoint, a versatile sampling framework minimizes costs and time for analyses, enhances precision and repeatability of experiments. Commonly applied sampling designs include the simple random sampling, stratified random sampling, or systematic random sampling. The simple random sampling considered a reference method, randomly selects calibration sites, irrespective of geolocations. Although simple random sampling is a relatively simple method, some parameters may be omitted or large data gaps appear in the sample. In contrast, stratified methods generate a set of homogenized sample groups precisely estimating the multidimensional distribution of chosen ancillary variables. For replicability in metrics, an unbiased estimate with the lowest errors is desirable.

### Data mining and predictive analysis

Futuristic models to support decision making utilize machine learning, data mining, and rule induction algorithms to decipher complex hierarchical relationships between predictors and response variables. These include the non-parametric yet parsimonious methods, such as artificial neural networks (ANNs), support vector machines (SVMs), principal component analyses (PCA), partial least squares regression (PLSR), genetic algorithms (GAs), and decision tree techniques (de Paul Obade and Moore 2018; Liou et al. 2004; Mehmood et al. 2012; Zeraatpisheh et al. 2020). Among the commonly used are decision trees which (i) handle non-parametric data, (ii) are robust against non-linearity and insensitive to missing data or outliers, and (iii) can utilize numerical, ordinal, binary, and categorical data (de Paul Obade and Lal 2013; Heung et al. 2014). Decision trees consist of leaf nodes and branches with each node representing a conditional statement, compartmentalized under the classification and regression tree. The classification tree generates a categorical outcome, whereas regression tree provides a continuous numerical outcome (Breiman et al. 1984). Random Forest (RF) is a modified ensemble of the Classification and Regression Tree algorithm (CART), incorporating “randomness” into its predictions through iterative bootstrap sampling, and is less susceptible to over-fitting (Heung et al. 2014; Zeraatpisheh et al. 2020). Comparatively, “bagging” aggregates the results of many trees, whereas boosting considers errors from previous classifier steps when sampling data for the next iteration (Breiman 2001).

### Geostatistical analyses and visualization

Geostatistical methods predict unknown point locations using observations made at neighboring positions, based on Tobler’s law which states that proximal observations or measurements are similar (Tobler 1970). Examples include local spatial averaging, Inverse Distance Weighing, and Kriging (Bilgili 2013; Goovaerts 1999). The local spatial average computes the value of unsampled locations from the mean of neighboring values; the problem being to define this local neighborhood. Comparatively, the Inverse Distance Weighting computes the values for unsampled locations as the weighted mean of neighboring values, with the weights decreasing linearly from the prediction location, the problem here being how to predict when distances are close to zero? In kriging, the linear model is fitted by ordinary least squares, and then a variogram is estimated for the residuals. On the other hand, co-kriging is a multivariate modification that combines a sparsely measured primary variable (or target variable) with a denser set of ancillary data considered as secondary variable (e.g., remote sensing data) to enhance accuracy (Odeh et al. 1995). Geostatistics methods support interpolation, spectral, spatial and temporal analysis useful for visualization and downstream scientific applications. However, issues remain, such as uncertainties arising from data gaps during scaling, and the requirement of dense point datasets (Davis 1987). Here, scaling refers to (i) the spatio-temporal resolution of phenomena or (ii) dimensions of the earth’s surface represented on paper and calculated as the ratio of the distance on a map, to the equivalent distance on the ground. Scale determines the (i) level of geoinformation detail extractable from a map and (ii) framework to audit environmental plans, which can either follow a “top-down” or “bottom-up” approach.

### Remote sensing in digital agriculture

The increasing data availability, computing power, and technical advances in remote sensing offer a unique opportunity to systematically monitor within-field soil quality dynamics. Remote sensing technology provides spatially continuous data even for inaccessible locations and distinguishes objects based on unique energy differences in the reflected (e.g., visible and Near Infra-Red), emitted (e.g., brightness temperature at Thermal Infra-Red), and backscattered (e.g., microwave) electromagnetic waves (de Paul Obade and Lal 2013; Khanal et al. 2017). Because soil is a multifunctional medium that is spatially heterogeneous, soil quality data is sensed in the field and laboratory using proximal sensors, following which information is gleaned and either upscaled or downscaled.

The visible (Vis) (400–780 nm), near-infrared reflectance (NIR) (780–2500 nm), short wave infra-red (SWIR), and thermal wavebands are single-band spectra convertible into band ratios or indices, to enhance signal and minimize soil background noise and solar irradiance (Liu et al. 2013). To extract information or develop soil quality diagnostic tools, indices can be integrated with laboratory measured soil properties (de Paul Obade and Lal 2013; Khanal et al. 2018). Optical-based reflectance spectroscopy has been used to estimate cation exchange capacity (CEC), available water content (AWC), soil organic carbon (SOC), base saturation, pH, exchangeable bases, and extractable phosphorus, clay content, extractable Fe, total elements, such as Ca, Mg, Fe, Mn, K, and Cu, and soil and plant health (Chen et al. 2019; Cohen et al. 2007; Minasny and Hartemink 2011; Sarkhot et al. 2011). Electromagnetic induction instruments attached on vehicles provide spatially referenced electrical conductivity estimates on soil mineralogy, salts, moisture, and texture. Dematte et al. (2007) found a high correlation between Landsat spectral reflectance data and soil texture, OC, and CEC.

Although remote sensors are non-destructive, fast, precise and relatively inexpensive, for acquiring data over large spatial extents, they only measure surrogate variables, thus require data integration, analyses, and visual inspection to glean information on the sensed data. Besides, optical remote sensors only acquire information from the top few millimeters (mm) of soil surface and are distorted by noise, such as surface roughness and moisture. Sundry details on sensor specifications, digital processing, and geometric and radiometric corrections are orthogonal to this work but are accessible online or from the following references (Chang et al. 2015; de Paul Obade et al. 2013; Dematte et al. 2007; Haji Gholizadeh et al. 2016; Huang et al. 2018; Khanal et al. 2020; Ouma 2016).

Generating accurate and reliable remote sensing products entails (i) fusion and mosaic to remove exposure differences and allow scale flexibility, and (ii) classification algorithms to map homogeneous attributes, for example, unsupervised that produces maps entirely from algorithms without prior knowledge or training datasets, or supervised classification based on training models using known sampled ground truth data. Other feature selection and separability algorithms, such as spectral mixture analysis (SMA), separate distinct objects. SMA decomposes spectra within pixels based on proportional cover of each pure class, or endmember, thereby enhancing clarity of map products. However, mapping soil characteristics require sensor signals that penetrate obstacles (e.g., soil depth, vegetative cover, or paved surfaces), or algorithms that indirectly predict soil property. Yet, the spectral, spatial, and temporal properties for detailed soil mapping are difficult to ascertain. However, because of soil spatial heterogeneity, SMA holds the promise of producing soil quality maps without disturbing the soil or landscape.

Among the issues to contend with in remote sensing applications include: (i) missing data in optical sensors mounted on satellite platforms arising from cloud cover, (ii) mixed signals arising from adjacency effects, topography and sun angle variation, viewing angle, atmospheric scattering, and absorption, (iii) scarcity of long-term datasets, or time relevance of data (i.e., sampling frequency and revisit time), and (iv) in the case of soil quality mapping, signal obstruction by buildings or vegetation, although changes in soil moisture or temperature, vegetation type, and health can serve as proxy indicators of soil quality (Huang et al. 2018; Kamilaris et al. 2017). Time series analyses with satellite imagery, though useful for monitoring, similarly experiences challenges, such as data gaps from cloud obscured pixels or shadowed pixels. These shortcomings can be minimized by using normalization algorithms, which screen out and merge the pseudo-invariant, i.e. temporally unchanged features on both the ground and imagery.

Table 1 outlines digital applications in soil quality assessment, with detailed review available in the following references (Kamilaris et al. 2017; Kamilaris and Prenafeta-Boldú 2018; Rossel et al. 2008; Rasouly et al. 2020). However, none of these used a single value SQI, although digital technology determined specific or individual soil properties (e.g., soil moisture, pH). Thus, the challenge remains interpolating a single value SQI developed by integrating qualitative data (e.g., management) with quantitative data (e.g., weather, vegetation, soil properties, and this information subsequently relayed instantaneously through cellular networks or internet).

**Table 1:** Overview of digital technology in soil quality assessment

Techniques	Key findings and limitations	Source
Disjunctive kriging (DK), ArcGIS, classification, kappa statistic	Soil salinity estimated However, soil salinity variables all skewed distribution and poorly correlated with terrain indices, though have strong correlations among each other	Bilgili (2013)
Indian Remote Sensing (IRS)-1B LISS-II digital data, field data and topographical maps, salinity indices (band combinations), supervised maximum likelihood classification	Over 65% of salt-affected soils found in shallow water table areas over 3 years	Abbas et al. (2013)
Soil Properties mapped using visible and near infrared (vis–NIR) reflectance spectroscopy technology (ASD FieldSpec Pro FR spectrometer). Spectral indices, Partial Least Squares Regression and Kriging	Only Soil Nitrogen mapped. However, the correlation between soil Nitrogen and other soil attributes e.g., Phosphates, Available water content and even SOC ignored	Liu et al. (2013)
Diffuse Reflectance Spectroscopy. Digital camera used to indirectly measure soil organic carbon (OC) and iron (Fe) contents using soil colour as the proxy. Predictions using univariate and full factorial regressions (FFR). visible-near infrared (vis–NIR: 400–1100 nm) spectra and partial least squares regression (PLSR)	Digital camera practically useful as a fast, accurate and non-destructive predictor of soil OC and Fe contents SOC predicted better than Fe However, interpolation of metrics unresolved Cellular networks not applied to transmit information in real time	Rossel et al. (2008)
3 Soil quality indices (SQIs) developed by quantifying several soil properties to discriminate effects of slope gradient and land use change on soil quality Soil quality indices maps were developed using digital soil mapping methods.	Steep slopes and geographic locations with land use conversion from grassland or forests to agriculture had lower soil quality This hypothetically is attributable to increased soil erosion, lower C input in croplands, or increased soil temperature and aeration enhancing mineralization	Nabiollahi et al. (2018)
Narrowband radio channel model application in wireless sensor networks for smart agriculture 3 radio frequencies used to distinguish soil, short and tall grass fields	Technique accurately distinguished soils from vegetation. However soil quality not evaluated. The technique together with information relayed are complex to be relayed in current format through cellular networks to stakeholders	Klaina et al. (2018)
Soil pH mapped using two machine learning techniques, namely Random Forest and XGBoos	Generated map provides pertinent information useful for: (i) Assessing impacts of changes in land use and climate on the soil’s pH, (ii) Guiding users on remediation and prevention of soil acidification, salinization and pollution by heavy metals, e.g., cadmium and mercury	Chen et al. (2019)
Principal component analysis used to screen out significant variables determining soil quality. Linear and non-linear scoring systems used to compute SQI Random forest technique used to generate soil quality map	(i) Results show that soils under natural forest were of a higher quality than soils under dry farming land use. (ii) The Linear scoring system had higher coefficient of determination (*R*^2^) with SQIs than the nonlinear scoring system	Zeraatpisheh et al. (2020)

## Case study

**Fig. 4:**
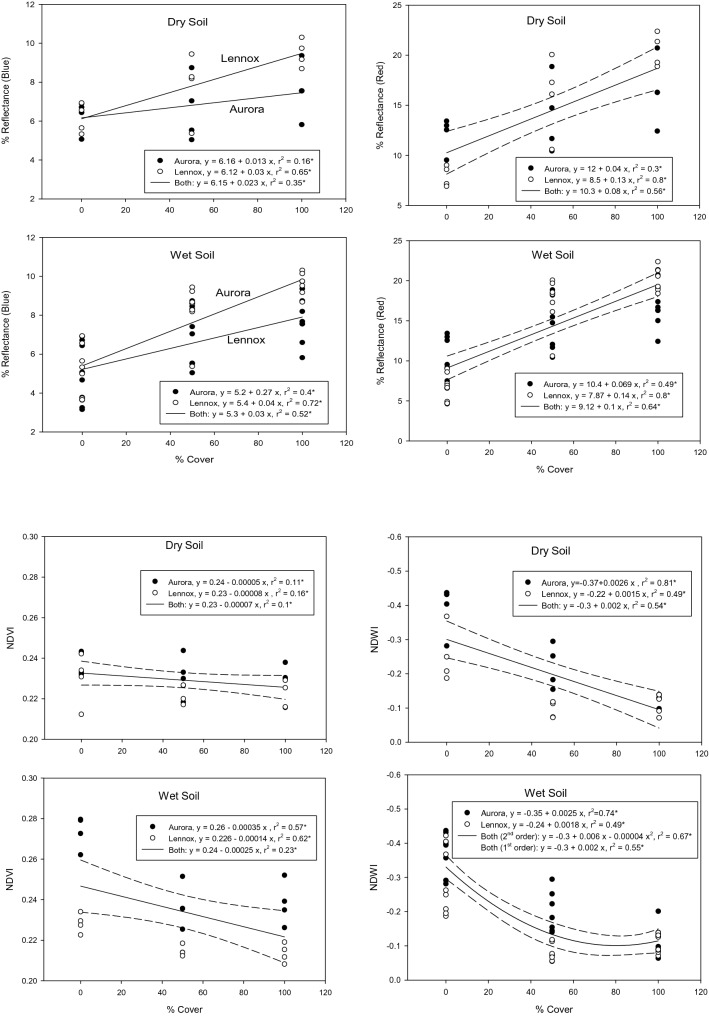
The interrelationship between surface residue cover on soils with varying soil moisture on blue and red spectral reflectance wavelength bands (top), Normalized Difference Vegetation Index (NDVI) and Normalized Difference Water Index (NDWI) (bottom). The data is from a study conducted at Aurora and Lennox site (South Dakota, USA). The soil moisture content approximately 8% for dry soil, and 20% for wet soil respectively. The 95% confidence interval for the regression equation is shown (Source: de Paul Obade (2011))

Regional assessments of surface residue cover remains work in progress despite the fact that crop residues play a principal role: (i) in replenishing soil nutrients, (ii) as alternative energy sources, (iii) in soil and water conservation, (iv) in sequestering C and regulating soil microclimate for biota to thrive. Traditional methods such as visual estimation and line transect are non-comprehensive over large areas due to gaps in measurements aggravated by time constraints. Advancements in computing systems and remote sensing enable large swaths of land, including inaccessible locations to be mapped, especially for homogeneous terrain/features, because heterogeneous surfaces generate mixed signals. Mapping surface residue cover in agricultural fields remains challenging because of the difficulty in separating spectral signatures of crop residues from bare soil, or standing vegetation. Figure 4 exemplifies the unique interrelationship between surface residue (corn (*Zea mays L.*) and soybean (*Glycine max* (L.) Merr.) cover on dry/wet soil vis-à-vis spectral reflectance based on a controlled experiment using data from Aurora site (44˚ 18′ 29″ North and 96˚ 40′ 13″ West), and Lennox site (43˚ 14′ 34″ North and 96˚ 14′ 0.9″ West), South Dakota, USA (de Paul Obade 2011).

To examine the influence of crop residue cover and soil wetness on spectral reflectance, 96 plots at Aurora site and 35 plots at Lennox site, each plot having a 2 m by 2 m dimension, were scanned under clear sky conditions with a handheld Cropscan multispectral radiometer (Cropscan Inc., Rochester, Minnesota, USA). Spectral  % reflectance measurements were taken at nadir with the radiometer set at a height of 2 m above the soil surface to approximate a 1 m^2^ ground spatial resolution, and calibrated by taking five spectral radiance readings on a standard reflectance white polyester tarp, before and after whole field had been scanned. The surface residue cover was measured using the line transect method, and a global positioning system (GPS) was used to geolocate the sampled plots. The soil types at Aurora site are fine-silty, mixed, frigid udic haploborolls (Munsell color chart reading of 10YR 4/2 and 10YR 5/3), whereas Lennox had fine-silty, mixed, mesic udic haplustolls (7.5YR 4/0 and 7.5YR 6/0). Surface soil data were randomly sampled before planting to a depth of 10 cm, and the moisture content was determined in the lab gravimetrically (Topp and Ferre 2002). Alternately, the correlation between percent surface residue and soil moisture vis-à-vis, the Normalized Difference Vegetation Index (NDVI), and Normalized Difference Water Index (NDWI) computed from ratios of spectral reflectance (*R*) at specific wavelength (nm) were determined, respectively:

1 $${\text{NDVI}} = \, \left( {R_{ 8 30} - R_{ 6 60} } \right)/\left( {R_{ 8 30} + R_{ 6 60} } \right),$$

2 $${\text{NDWI }} = \, (R_{ 8 30} - R_{ 1 6 50} /\left( {R_{ 8 30} + R_{ 1 6 50} } \right).$$

According to Fig. 4, the sensitivity of individual bands and the indices (i.e. NDVI and NDWI) varied significantly with % residue cover at specific fields, suggesting that soil water content impacts site specific spectral reflectance. However, these results should be interpreted with caution because decaying residue also contribute to variability in spectral reflectance. Proximal or ground based sensors (e.g., Cropscan) suffice for insitu acquisition of spectral signatures of heterogeneous features such as soil properties or % surface crop residue cover that are challenging to scan from high altitude. Ground based sensors generate data with higher signal to noise ratio (SNR) (i.e., less errors) attributed to less atmospheric attenuation of signals because signals travel over-shorter atmospheric path length, compared with sensors on-board aerial or satellite platforms, whereby haze, cloud cover, and atmospheric scattering, attributed to the high altitude, generate substantial errors.

### Pedotransfer functions (PTFs)

Although digital technology processes big data simultaneously, intensive acquisition of field data, laboratory testing, and analyses necessary for validation can be prohibitively expensive (de Paul Obade and Lal 2013). In situations where data on specific soil properties are unavailable or expensive to measure, these properties may be predicted using pedotransfer functions (PTF) (Hartemink 2008; McBratney et al. 2011; Tranter et al. 2009). Documentation exists on PTFs providing proxy values: (i) predicting Phosphorus (P) sorption and fixation; (ii) estimating bulk density, particle size, and SOC (Calhoun et al. 2001); and (iii) estimating soil water retention for specific topography, geographic location, and class horizons (McBratney et al. 2011; Rawls and Brakensiek 1982; Rawls et al. 2003; Tranter et al. 2009). The equations in Fig. 4 exemplify PTFs for estimating surface residue cover using remote sensing under wet and dry soil moisture conditions.

Multivariate models routinely used in PTFs include linear regression, generalized linear models (GLIM), generalized additive models (GAM), neural networks (NN), support vector machines (SVM), decision trees (i.e., classification, regression trees, and random forest). Otherwise, linear regression though frequently used, is ineffective for synthesizing soil quality metrics because of multicollinearity issues related to soil heterogeneity, and non linear relationship between the numerous soil property response and predictor variables. In light of this, fewer parameters or “minimum dataset selection,” a feat attainable through data aggregation and/or reduction using nonlinear machine learning methods, such as NN, genetic algorithms, random forest, multivariate adaptive regression splines, and principal component analyses (PCA), suffice. PCA generates new significant variables or components from original datasets by projecting each data point onto a linear combination of the variables. However, PCA may be imprecise when the dataset contains a high percentage of missing values, whereas NNs, which mimic interconnected biological nodes or neurons, are complex black boxes, thus difficult to decipher the meaning of information. SVM constructs sets of hyperplanes in an infinite-dimensional space separated by linear, radial, sigmoid, or polynomial kernel functions (Khanal et al. 2018). Because PTFs are applicable in filling data gaps, they are a key solution for interpolating the spatio-temporal SQI variability (de Paul Obade 2019). However, models being imperfect operate under assumptions, thus should be interpreted with caution (de Paul Obade and Lal 2014a, b; Minasny and Hartemink 2011; Shepherd and Walsh 2002). Other considerations include ascertaining the credibility of SQI by validating with metrics such as soil biota (e.g., respiration, earthworm density, microbial biomass, etc.) which are not only sensitive to environmental gradients but also play a central role in soil functioning.

## Assessing information efficacy

A key challenge when interpreting information is quantifying its currency, accuracy, and explanatory power. This is especially so regarding soil properties which are spatially heterogeneous. Any measurement is prone to errors, which harkens back to the debate on whether information from digital technology will be credible for effecting best management soil quality practices. Otherwise, from a technical perspective, regression models evaluate the “goodness of fit” between predicted and actual values, with proportion of information in the data explained by the model quantified using correlation analysis or coefficient of determination (*R*^2^). Usually the data are split into calibration and validation sets, proportionately for instance, in a ratio of 3:1, to statistical quantify uncertainty. Model “fit” is quantified using *R*^2^, mean error (ME), and the root mean square error (RMSE) with a high *R*^2^, small RMSE, or ME suggesting higher correlation between predictor and actual in situ data (Davis 1987; Khanal et al. 2018). Similarly, the Pearson correlation coefficient “r,” has values ranging from − 1 to + 1, with a positive “*r*” value indicating a positive association, with 1.0 as maximum, whereas 0 denotes no association between variables. The accuracy of remotely sensed information is evaluated through the error matrix or contingency table which compares the ratio of the correctly classified pixels (sum of diagonal number of pixels in the *matrix*) to the total number of classified pixels, whereas Kappa Index evaluates the probability of a chance classification for a specific pixel (Congalton 1991).

## Conclusion

This contribution is a synopsis of issues surrounding the adoption of digital technology as decision support tools for judiciously managing and optimizing agronomic input while reducing environmental footprints. Although an evolving science, digital technology creates opportunities to pinpoint potential areas of concern, experiment, and develop new objective metrics that could not only offer scientific information for strategies geared towards enhancing net biome productivity, water, and nutrient use efficiencies but also a tracking mechanism for assessing environmental compliance of land use practices. The challenge remains relaying credible scientific information instantaneously and, in a format, understandable to end-users. For agricultural applications, the SQI information should be comprehensive yet clear with minimal abstraction. However, because of absence of universal SQI, the SQI metrics should be interpreted cautiously with local tacit and expert knowledge to avoid making false assumptions or conclusions. Other research prospects related to SQIs include (i) quantifying environmental footprint vis-à-vis climate change trends on agricultural systems; (ii) assessing the threshold of natural habitats to sustain ecosystem services; and (iii) quantifying the value addition of investment on digital technologies for SQI mapping.

## Data Availability

Not applicable.

## Abbreviations

AWC: Available water content
CART: Classification and Regression Tree algorithm
CEC: Cation Exchange Capacity
GIS: Geographical Information Systems
GNSS: Global navigational satellite systems
GPS: Global positioning system
GLIM: Generalized linear models
GAM: Generalized additive models
IR: Infra-red
ME: Mean error
NIR: Near Infra-Red
NDVI: Normalized Difference Vegetation Index
NDWI: Normalized Difference Water Index
NN: Neural networks
P: Phosphorus
PTF: Pedotransfer functions
PCA: Principal component analyses
SWIR: Short-Wave Infra-Red
SVM: Support vector machines
TIR: Thermal Infra-Red
RF: Random Forest
RMSE: Root Mean Square Error
SOC: Soil organic carbon
SOM: Soil organic matter
SQI: Soil quality index
SMA: Spectral mixture analysis
UAV: Unmanned aerial vehicle
U.N.: United Nations
VRT: Variable rate technologies
